# Phytoplankton Composition and Abundance in Restored Maltański Reservoir under the Influence of Physico-Chemical Variables and Zooplankton Grazing Pressure

**DOI:** 10.1371/journal.pone.0124738

**Published:** 2015-04-23

**Authors:** Anna Kozak, Ryszard Gołdyn, Renata Dondajewska

**Affiliations:** Department of Water Protection, Faculty of Biology, Adam Mickiewicz University, Poznań, Poland; Stazione Zoologica Anton Dohrn, ITALY

## Abstract

In this paper we present the effects of environmental factors and zooplankton food pressure on phytoplankton in the restored man-made Maltański Reservoir (MR). Two methods of restoration: biomanipulation and phosphorus inactivation have been applied in the reservoir. Nine taxonomical groups of phytoplankton represented in total by 183 taxa were stated there. The richest groups in respect of taxa number were green algae, cyanobacteria and diatoms. The diatoms, cryptophytes, chrysophytes, cyanobacteria, green algae and euglenophytes dominated in terms of abundance and/or biomass. There were significant changes among environmental parameters resulting from restoration measures which influenced the phytoplankton populations in the reservoir. These measures led to a decrease of phosphorus concentration due to its chemical inactivation and enhanced zooplankton grazing as a result of planktivorous fish stocking. The aim of the study is to analyse the reaction of phytoplankton to the restoration measures and, most importantly, to determine the extent to which the qualitative and quantitative composition of phytoplankton depends on variables changing under the influence of restoration in comparison with other environmental variables. We stated that application of restoration methods did cause significant changes in phytoplankton community structure. The abundance of most phytoplankton taxa was negatively correlated with large zooplankton filter feeders, and positively with zooplankton predators and concentrations of ammonium nitrogen and partly of phosphates. However, restoration was insufficient in the case of decreasing phytoplankton abundance. The effects of restoration treatments were of less importance for the abundance of phytoplankton than parameters that were independent of the restoration. This was due to the continuous inflow of large loads of nutrients from the area of the river catchment.

## Introduction

The eutrophication of water bodies accelerated by human activity creates the need for various activities to be undertaken to restore good water quality. Phytoplankton, as a very reactive element of the lake ecosystem, is first to react to changes in the environment. These changes concern both quantitative and qualitative phytoplankton structure [1–4]. Considerable changes in the phytoplankton community were reported from the restored Lake Trummen. After removal of the top sediment layer the species diversity of phytoplankton increased and phytoplankton biomass decreased in the lake [5]. At the same time the actual plankton community disappeared (forms such as cysts, spores, zygotes, hormogonia etc. were removed with the sediments). The costs of this removal are the most important disadvantage in applying this method as it is several times more expensive than P-inactivation [6]. Chemical treatment, which seems to be one of the most popular restoration methods used on lakes, results in rapid and satisfactory effects [7–15]. Owing to their effectiveness for phosphorus inactivation, aluminum and/or iron, salts have most often been used as coagulants applied in lakes and reservoirs during restorations [1]. Other compounds can also be used to precipitate phosphorus from the water column or for inactivation in bottom sediments, for example Phoslock, Sinobent, nitrates, magnesium chloride, etc. [10]. The immediate effect of an application of Phoslock causing a decrease of phytoplankton abundance, especially cyanobacteria, was stated in the Laguna Niguel Lake in California. Cyanobacteria-dominated blooms at high densities were replaced by diatoms and green algae soon after the application of Phoslock [16]. Sometimes one method of restoration treatment is insufficient to cause a decrease of phytoplankton growth [17]. A combination of two methods seems to be more effective, e.g. simultaneously applied flocculent polyaluminium chloride and Phoslock turned out to be more effective in reducing the cyanobacterial bloom caused by *Microcystis aeruginosa* and *Aphanizomenon flos-aquae* in Lake Rauwbraken, located in the south of the Netherlands [18]. Diatoms, also a very sensitive group, was replaced by another, green algae, soon after a Phoslock application [19]. However, the responses of phytoplankton to restoration treatments are not always are so spectacular. Reports from the restored Lake Balaton stated that the response of phytoplankton was not easily visible and sometimes counterintuitive [20].

Chemical methods cause substantial changes in the whole ecosystem and require large amounts of chemicals [18]. Such a treatment, with tonnes of chemicals dosed into the water, was not acceptable in the case of Maltański Reservoir. Therefore, sustainable treatment was adopted there with a small amount of coagulant being applied several times a year. This is a new approach to the restoration of lakes, and its impact on phytoplankton is not yet well documented.

This study focused on phytoplankton dynamics under the influence of changes in phosphorus concentration due to its inactivation, as well as zooplankton grazing due to implemented biomanipulation measures. The influence of these variables was also compared with the impact of other environmental variables to assess to what extent the restoration methods are effective in limiting the growth of phytoplankton in the reservoir.

## Materials and Methods

Maltański Reservoir (52°24′N, 16°58′E) is located in the central part of Poznań, mid-western Poland. It was created in 1952 by damming the waters of the River Cybina. It has a maximum depth of ca 5 m. Its area is 64 ha, average depth 3 m and a volume of 2·10^6^ m^3^. It accumulates water from the lower course of the River Cybina, the right tributary of the River Warta. The catchment area of the River Cybina represents a typical agricultural region, farms and fields dominate there. The reservoir is intensely used for recreation and for water sports, and sport events of significant international importance take place there. It has been completely drained at 4-years intervals since autumn 1992 and filled with water early in the following spring so as to carry out maintenance of the weir and sports facilities. The water quality of the reservoir, since its filling with water in 1993, has been hypertrophic. Therefore it was restored for the first time in the years 1993–96 by means of biomanipulation. The reservoir was stocked with predatory fish (pike *Esox lucius* (L.), pikeperch *Stizostedion lucioperca* (L.) and wels catfish *Silurus glanis* L.) and an increase of water transparency was expected due to a cascading top-down impact on phytoplankton density [21–22]. However, the effectiveness of stocking was not sufficient as so-called feed back effects appeared [23–24]. Phytoplankton abundance during the application of the biomanipulation method was low only at the beginning of the experiment; in subsequent years dominating colonial cyanobacteria and permanent water bloom during summer seasons made the recreational use of this reservoir impossible [17]. Therefore, a second method was implemented from 2005 to strengthen the restoration treatment [25]—this time iron sulphate (PIX) was added to the reservoir in small doses.

During this study, in the period of 2009–2012, juvenile fish of species such as pikeperch, pike and wels catfish were stocked in Maltański Reservoir, especially fry and summer and autumn fingerlings (Wiśniewski, unpubl.). Approximately 600–800 kg per 1 ha every year were introduced in the period of 2009–2011. The reservoir was not stocked with fish in 2012 because it was the last year before its draining. During the draining at the end of 2012 all the fish were caught and removed from the reservoir. The most abundant species were bream *Abramis brama* (L.)- 47%, roach *Rutilus rutilus* (L.)- 15% and perch *Perca fluviatilis* (L.)- 14%. Altogether, there were 21,442 kg of fish. Phosphorus inactivation was undertaken using small doses of coagulants several times a year. Three coagulants were dosed there: PIX, Sinobent and magnesium chloride. PIX was dosed 9 times a year in 2009, 6 times in 2010 and 2011, and 3 times in 2012. Each time the dose of the coagulant amounted to 500–700 kg for the whole reservoir (about 10 kg ha^-1^). Additionally, once in 2011 and three times in 2012 a dose of 150 kg of a new coagulant Sinobent [10] was added into the reservoir. 300 kg of MgCl_2_ were also added there 3 times in 2012.

This paper includes the results of a 4-year-long series of studies of plankton and physico-chemical characteristics of water, from the period beginning in 2009 when Maltański Reservoir was filled with water till its drainage in 2012. The composition and abundance of nine phytoplankton groups and zooplankton, i.e. cladocerans, copepods and rotifers was analysed.

Phyto- and zooplankton composition as well as selected water features were analysed in 2 week intervals, mostly from March to October, from the surface and from the depth of 1, 2 and 3 m. Each time the material was collected using a 5 L sampler. Phytoplankton samples were collected without filtration. For zooplankton analyses 10 l of water was filtered through a plankton net, mesh size 30 μm. The water temperature, concentration of dissolved oxygen, conductivity and pH were measured directly in the field using a WTW 350 multi-parameter meter. Secchi disk visibility was also measured. Physico-chemical and biological parameters, such as concentration of nitrogen (ammonium, nitrite, nitrate, organic and total N) and phosphorus (dissolved phosphates and total phosphorus) were analysed in the vertical profile of the lake. Concentration of nitrogen and phosphorus forms and chlorophyll-a were analysed spectrophotometrically according to standard methods [26].

Samples of phyto- and zooplankton were fixed with Lugol solution. Phytoplankton composition and abundance was analysed using an Olympus microscope. The specimens (spec.) were counted in a Sedgwick-Rafter chamber of a volume of 0.67 ml for phyto- and 1 ml for zooplankton. Phytoplankton biomass was calculated by approximating the shape of specimens with geometric figures [27]. Crustacean biomass was calculated acc. to Bottrell et al. [28] based on the relationship between body length and body weight for each crustacean species. Rotifer biomass was taken after Ejsmont-Karabin [29] and Radwan [30]. The influence of zooplankton on individual taxa of phytoplankton as well as on the size of groups and taxonomical groups was analysed. For this purpose the phytoplankton was divided into two size fractions i.e. nanophytoplankton (up to 30 μm) and the microphytoplankton (above 30 μm).

Indicators of the specific Shannon–Weaver diversity index [31] of the phytoplankton and evenness index were counted using the PAST program (http:folk.uio.no/ohammer/past/).

Differences between phytoplankton abundance, biomass and its biodiversity (Shannon-Weaver and evenness indexes) of the individual studied years were tested by the non-parametric ANOVA Kruskal-Wallis Rank test (p < 0.05, PAST program). In order to ascertain the environmental variables in determining the abundance and biomass of phytoplankton the Canonical Correspondence Analysis was applied [32]. The appropriateness of using RDA was checked using DCA. Redundancy analysis (RDA) was used to create models explaining relationships between phytoplankton and physico-chemical parameters and zooplankton food pressure. The phytoplankton (2 size groups, nine taxonomic groups and individual taxa appearing in high density >500 spec. ml^-1^) was correlated with the parameters directly connected with restoration and independent of restoration treatment in order to determine which of them have a significant impact.

Only biomass (both of phyto- and zooplankton) was chosen for graphic illustration in the present paper. As zooplankton biomass is correlated with filtering rate and grazing rate [33–34], it perfectly reflects the alimentary pressure of the zooplankton on the phytoplankton. Testing correlations, zooplankton was divided into 3 groups: predators (mostly copepods: cyclopoids, elder larval forms of copepodites and *Leptodora kindtii* from Cladocera), filter feeders (cladocerans without *L*. *kindtii* together with calanoids from Copepoda) and small zooplankton (rotifers and nauplii—the young larval copepod forms). Such a division was necessitated due to two main factors influencing zooplankton: firstly—their feeding habits, and secondly—their exposure to the feeding pressure of fish (as biomanipulation, as a result of fish pressure, caused changes in zooplankton abundance and biomass).

The analysed parameters influencing phytoplankton which were directly connected with restoration procedures were tested separately from physico-chemical parameters that were independent of restoration. There were 4 parameters in the first group, i.e. filter feeders (mostly cladocerans) and predators (mostly cyclopoids) connected with biomanipulation (directly under food pressure of fish) and concentration of PO_4_-P and NH_4_-N, associated with chemical treatments, i.e. iron sulphate and magnesium chloride. The results obtained from all the sampling depths in the vertical profile were tested. The Monte Carlo permutation test with 499 unrestricted permutations of the constraining variable was used to assess the statistical significance in the regression.

The work did not involve any endangered or protected biological species. No specific permissions were required for these locations and activities.

## Results

### Physical and chemical variables

The highest values of Secchi depth (SD) in the MR during the studied period were stated in the first year of the study (after filling the reservoir with water). The maximum value of 3.8 m was found in May 2009 (Table 1). Between 2010 and 2011 a clear reduction of Secchi depth was visible. Throughout the whole of 2010 it did not exceed 1.65 m (June) and 1.2 m in 2011 (April). A clear improvement was found in 2012 and the transparency increased to 3.3 m (May). The water temperature in consecutive seasons reached its highest values in the summer months and ranged from 24.2 °C (2012) to 24.9 °C (in 2010) in the studied period (Table 1). The lowest temperature in summer was found in the last year of the study.

**Table 1:** Range of physico-chemical data of Maltański Reservoir.

Variables	mean	min	max	SD
**SD (m)**	1.11	0.45	3.3	0.53
**temperature (** ^**°**^ **C)**	16.97	6.32	24.9	4.76
**conductivity (μS cm** ^**-1**^ **)**	634	455	786	75
**pH**		7.30	9.38	
**dissolved oxygen (mg O**_**2**_ **l** ^**-1**^ **)**	8.98	0.35	16.03	3.24
**oxygen saturation (%)**	92.1	3.9	179.0	32.05
**seston mg l** ^**-1**^	11.7	1,0	31.1	5.74
**total N mg l** ^**-1**^	2.780	1.262	6.601	1.22
**total P mg l** ^**-1**^	0.130	0.015	0.68	0.08
**BOD**_**5**_ **mg O**_**2**_ **l** ^**-1**^	6.26	1.16	48.05	4.29
**chlorophyll a μg l** ^**-1**^	30.34	0.86	146.1	27.88

The oxygen saturation of water ranged from 4 to 179%. In all the years, good oxygen saturation in the water was observed at almost all depths from the surface to the bottom. Only occasionally did dissolved oxygen content in water decrease to 0.4 mg l^-1^ at the depth of 3 m (in August 2011) which accounted to only 4% of the oxygen saturation (Table 1). BOD_5_ varied from 1.2 to 48.1 O_2_ mg l^-1^ (Table 1). Electrolytic conductivity in subsequent years underwent a gradual decrease. The mean value amounted to 635 μS cm^-1^ (Table 1). The pH value varied in the analysed period between 7.3 and 9.4. Both the concentration of chlorophyll a and dry weight of seston showed the highest values in 2011.

### Nutrients

**Fig 1:**
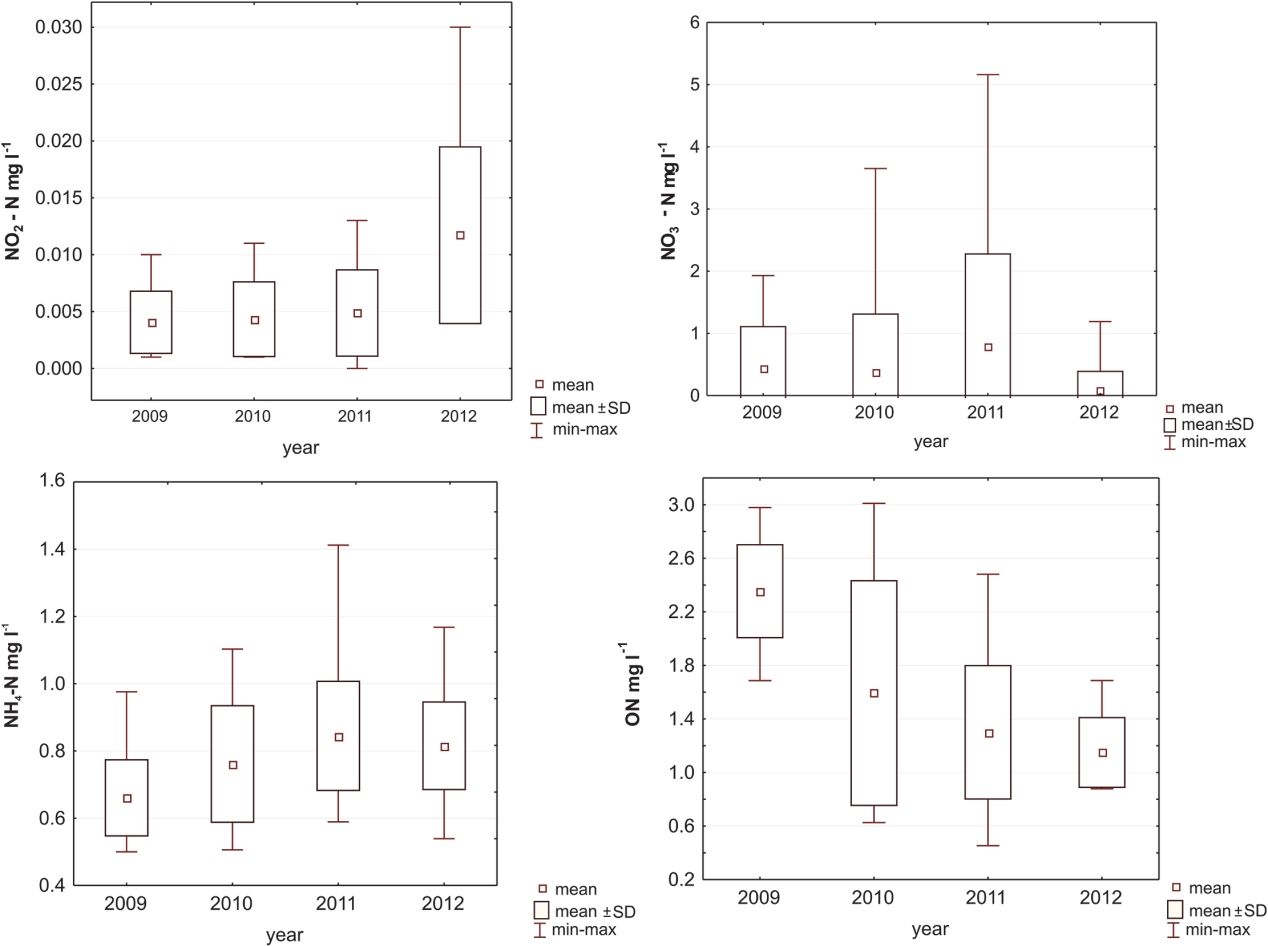
Concentration of nitrogen forms in Maltański Reservoir.

Values of ammonium nitrogen varied between 0.5 mg l^-1^ NH_4_-N and 1.41 mg l^-1^ NH_4_-N in the studied period. The highest concentration was noted in August 2011 (Fig 1). High values were noted in May 2009 (0.91 mg l^-1^ NH_4_-N), in August 2010 (1.10 mg l^-1^ NH_4_-N) and in September 2012 (1.03 mg l^-1^ NH_4_-N). Nitrite concentration increased from April to June. Much lower nitrite concentrations were observed from July to September (the highest in the studied period was noted in 2012, 0.03 mg l^-1^ NO_2_-N). Nitrate concentration was lower in summer and autumn. The highest values were noted every studied year in spring with maxima in April (maximum value 5.16 mg l^-1^ NO_3_-N in 2011).

The concentration of total nitrogen was high from April to May and usually significantly decreased from June or July to the end of the year. Average values of the year showed a downward trend in subsequent years.

**Fig 2:**
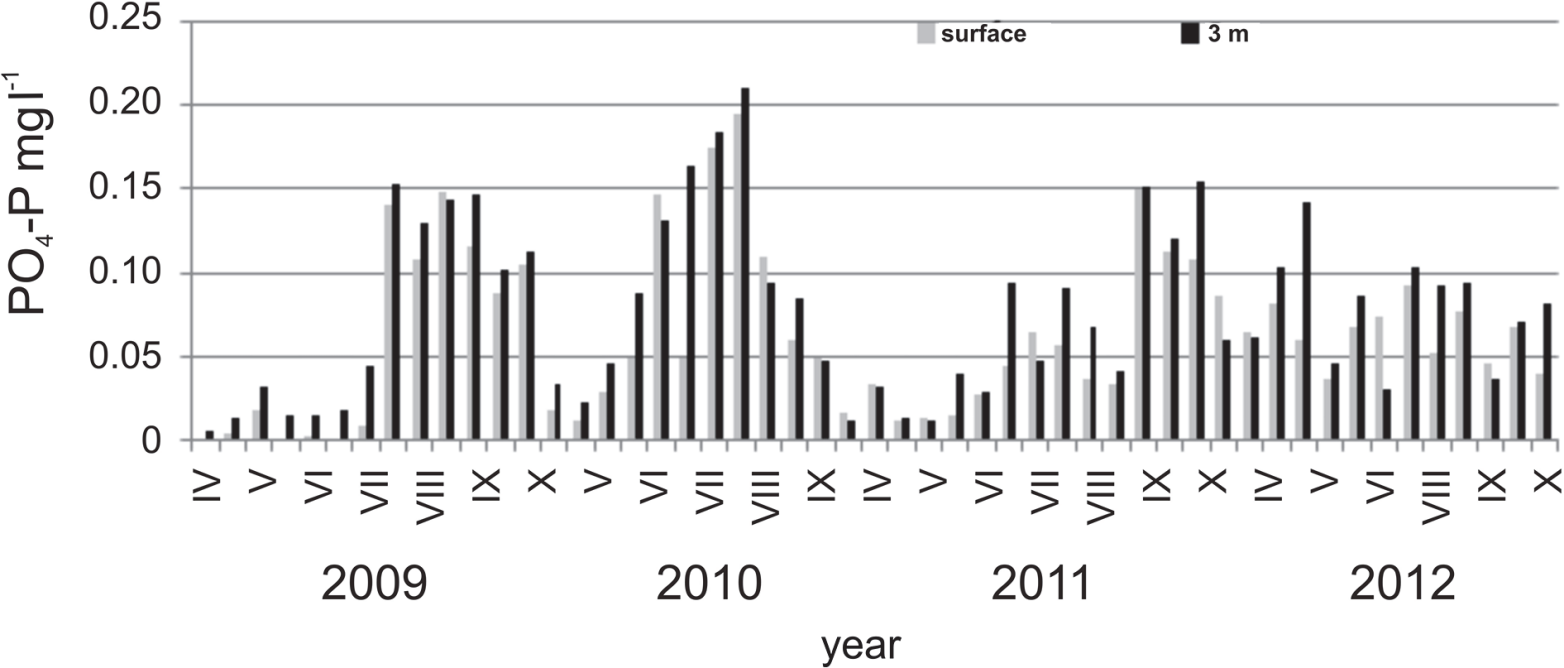
Concentration of phosphate phosphorus on the surface and the depth of 3m.

Higher concentrations of phosphates were recorded in the first two years of the study (especially 2010), the highest value was found in August 2010 (0.21 mg l^-1^ P, Fig 2). An upward trend in phosphorus levels in the water was noted in subsequent years. The highest concentration of total phosphorus was found in October 2011.

### Phytoplankton

**Fig 3:**
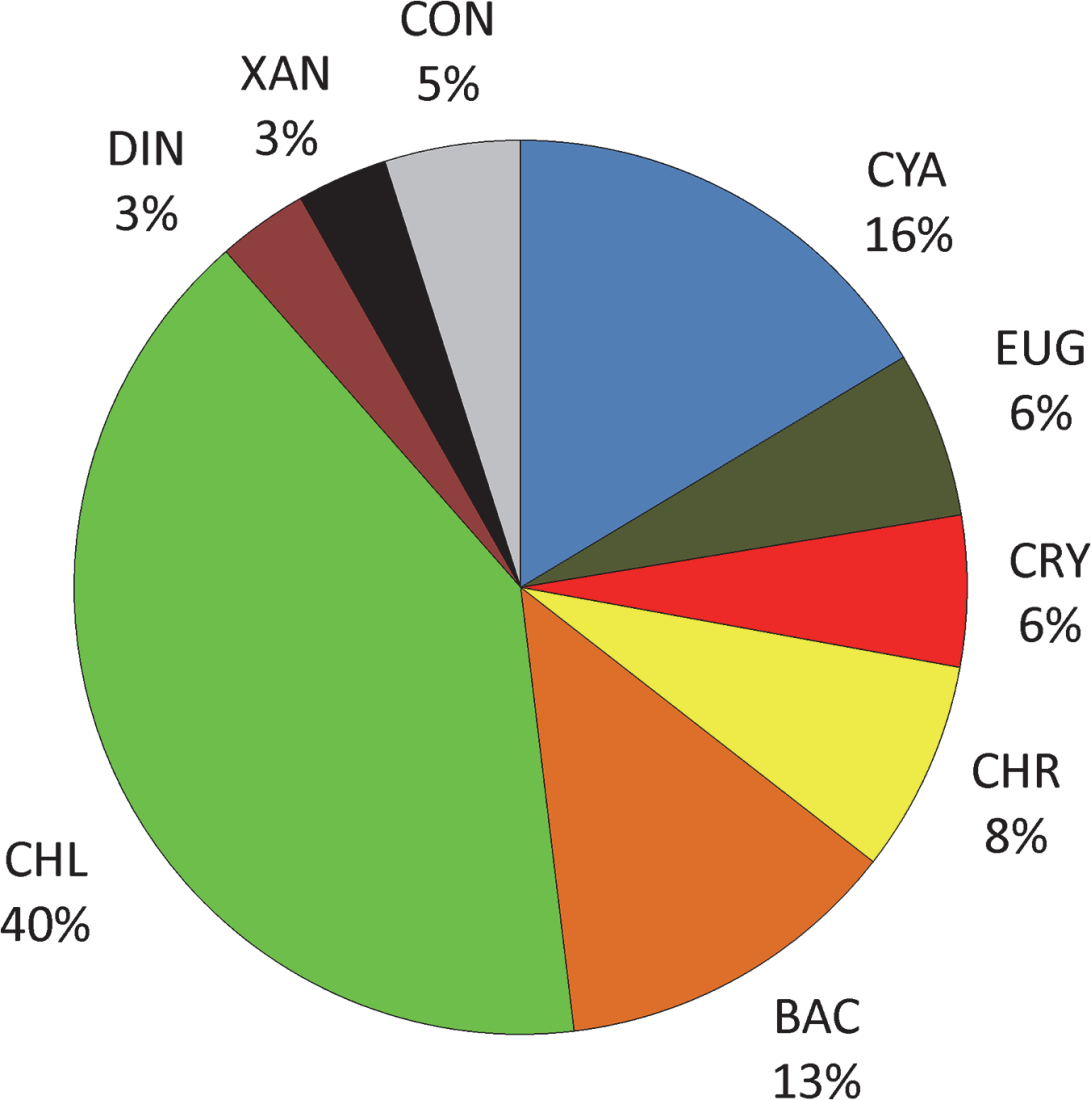
The participation of taxa belonging to main algal groups identified in Maltański Reservoir. CYA-Cyanobacteria, EUG-Euglenophyceae, CRY-Cryptophyceae, CHR-Chrysophyceae, BAC-Bacillariophyceae, CHL-Chlorophyceae, DIN-Dinophyceae, XAN-Xantophyceae, CON-Conjugatophyceae.

183 taxa representing nine taxonomic groups of phytoplankton were recorded in the studied reservoir. Green algae (74 taxa), cyanobacteria (30) and diatoms (23) were richest in taxa (Fig 3). Other groups were represented by a smaller number of taxa (6–14). In subsequent years the number of taxa ranged from 128 in 2009 to 156 in 2010.

**Fig 4:**
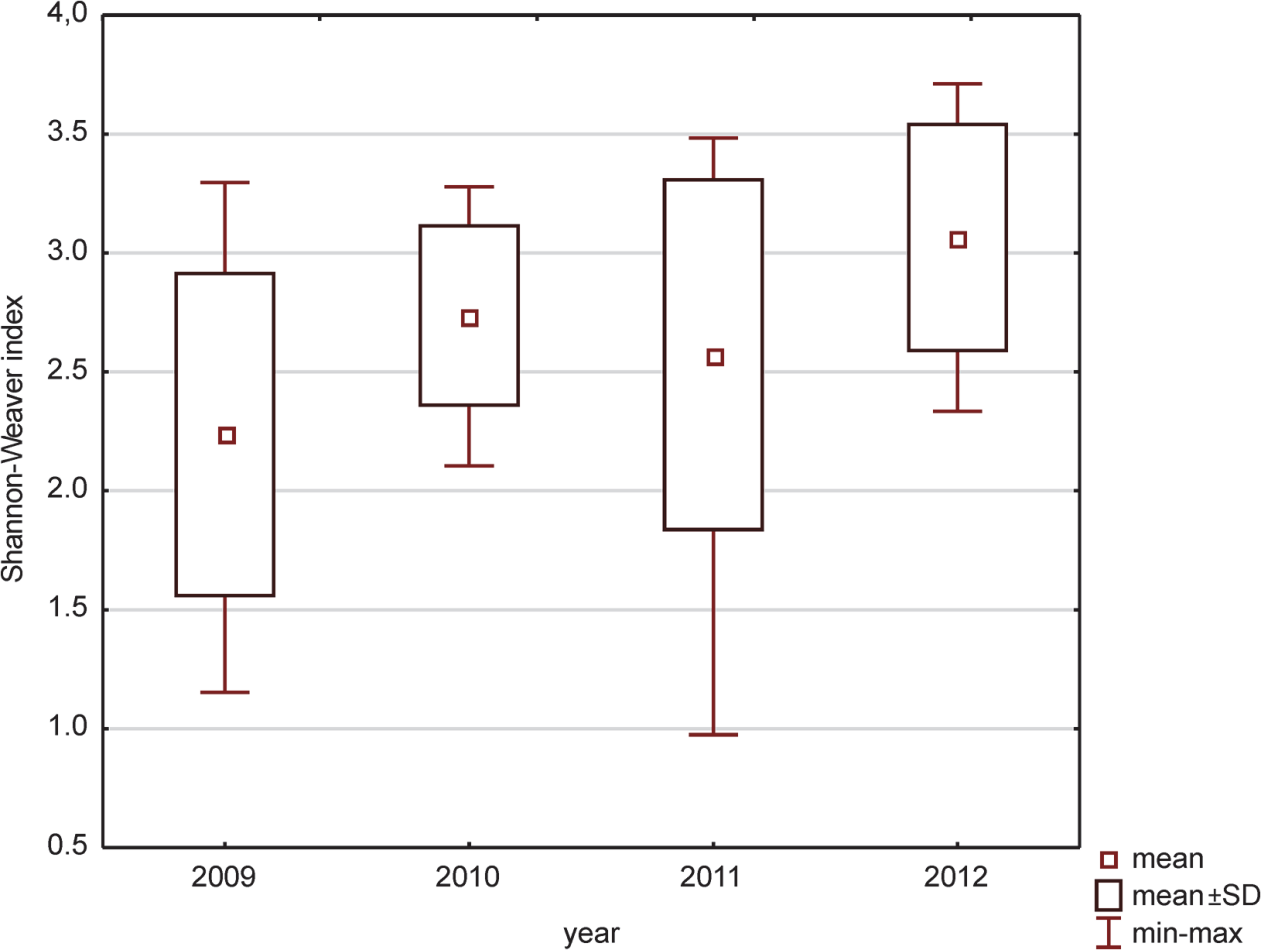
The mean, maximum and minimum values of Shannon-Weaver index in each year in Maltański Reservoir.

The highest value of the Shannon–Weaver diversity index was observed in 2012 (3.71, Fig 4). The average values of this index for each year were lowest in the first year of the study (2.25 in 2009). The value reached 2.71 and 2.58 in next two years and the highest mean value in the fourth year of the study (3.07 in 2012). Differences in values of Shannon–Weaver diversity index between the individual years were significant in most cases (p < 0.01, ANOVA Kruskal-Wallis test). Only the differences between the 2011 value versus other years were not statistically significant.

**Fig 5:**
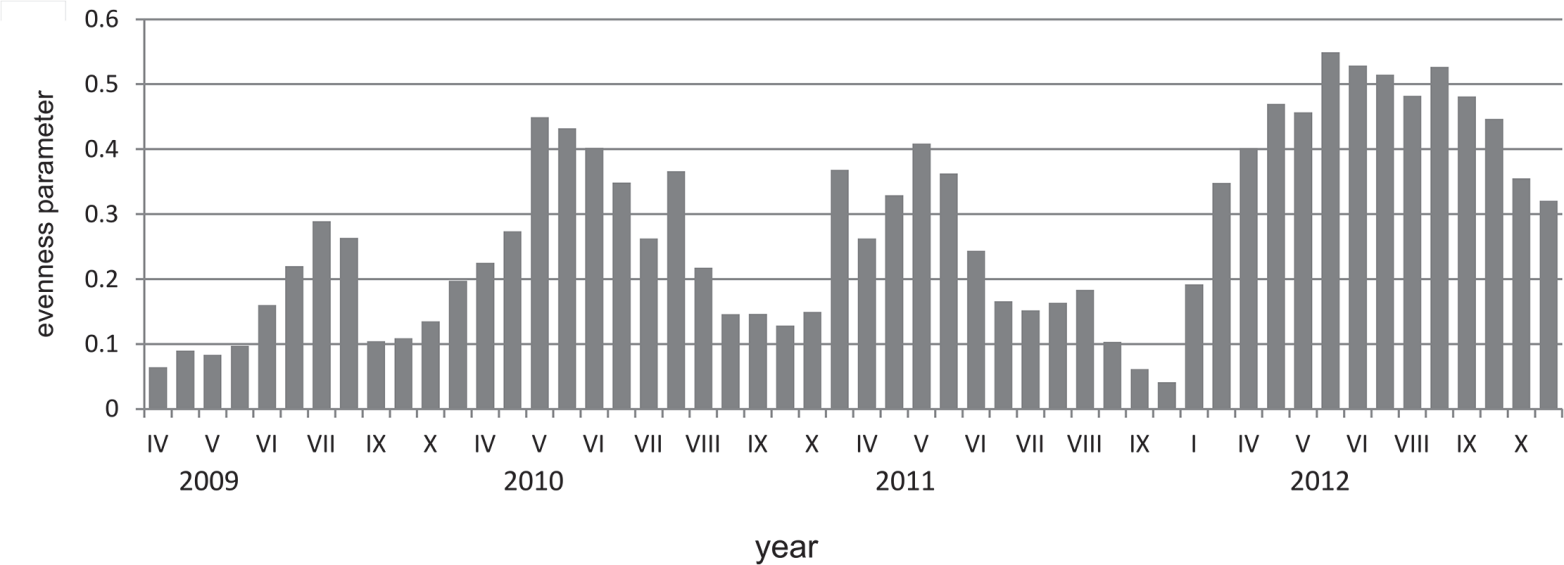
The diversity of evenness parameter in each sampling date in Maltański Reservoir.

The mean values of the evenness index in subsequent years were similar in first two years (0.28 and 0.27), while the highest value was noticed in 2012. The lowest value was reached in October 2011 (0.04), while the highest in June 2012 (0.55, Fig 5). The differences between the values in every studied year were statistically significant (p < 0.01, ANOVA Kruskal-Wallis test).

**Fig 6:**
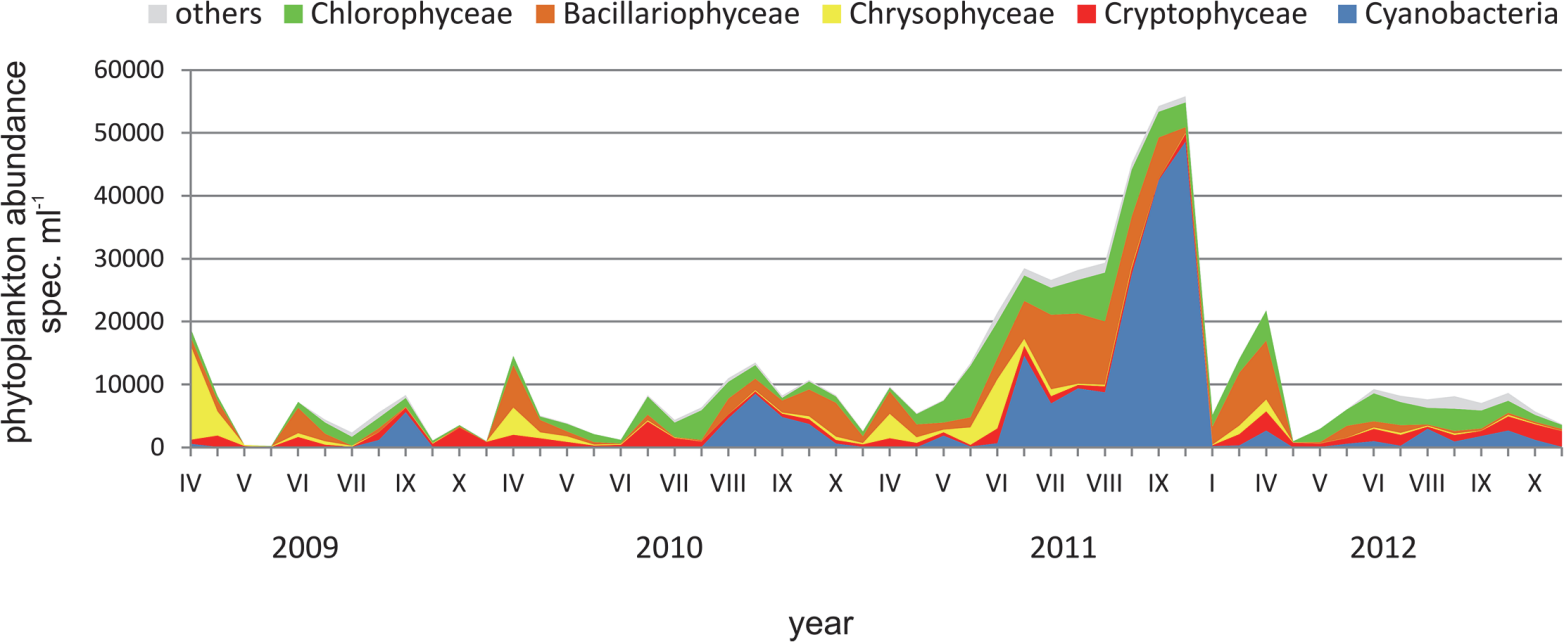
The abundance of phytoplankton groups in the investigated period (an example from the depth of 1 m).

Phytoplankton abundance in each of the sucessive years of the research evolved with the highest values in April (2009 and 2010) or September (2011 and 2012). Comparing consecutive years, the highest abundance of phytoplankton specimens was found in 2011 60·10^3^ spec. ml^-1^ (Fig 6). The highest values of the remaining years of the study were significantly lower (29.0 10^3^ spec. ml^-1^ in 2009 and ranging 10.6–14.2 10^3^ spec. ml^-1^ in 2010 and 2012). A clear decline in phytoplankton was recorded in late spring and summer (the so-called clear water phase established in May 2009 and 2012), when the abundance of phytoplankton decreased to 350 spec. ml^-1^ in 2009 and 550 spec. ml^-1^ in 2012 (the average value of the depth profile).

Seasonal changes in the dominance of particular groups of phytoplankton were found in subsequent years of the study. Chrysophytes (especially in 2009), cryptophytes, diatoms and chlorophytes dominated in phytoplankton in the period from April to May. The species that were the most abundant in phytoplankton during this period were *Erkenia subaequiciliata* Skuja, *Dinobryon sociale* (Ehrenberg) Ehrenberg, *Chrysococcus* sp., *Cryptomonas marssonii* Skuja, *Rhodomonas lacustris* Pascher & Ruttner, *Desmodesmus communis* (E.Hegewald) E.Hegewald, *Schroederia setigera* (Schröder) Lemmermann, *Tetraedron minimum* (A.Braun) Hansgirg, *Ulnaria acus* (Kützing) M.Aboal and *U*. *delicatissima* var. *angustissima* (Grunow) M.Aboal & P.C.Silva. Diatoms and green algae increased their share in the density in June. Then, from July to August or September a gradual increase in the share of cyanobacteria in the total abundance of phytoplankton was recorded. High abundance of this group was found especially in 2011. Its number increased gradually from July to October. The share of cyanobacteria in the studied period was the smallest in 2012, when it did not exceed 40%, while the maximum share in the period 2009–2011 ranged from 55–85%. The highest share of cyanobacteria in the studied period was recorded in September. Due to temperature decrease in October, the phytoplankton density significantly decreased and the share of cyanobacteria was lower. Exceptionally in 2011 cyanobacteria also dominated in abundance in October. The most numerous species of this group in subsequent years were *Pseudanabaena limnetica* (Lemmermann) Komárek, *Planktolyngbya limnetica* (Lemmermann) J.Komárková-Legnerová & G.Cronberg (especially in 2011), *Aphanizomenon gracile* (Lemmermann) Lemmermann and *Limnothrix redekei* (van Goor) Meffert.

**Fig 7:**
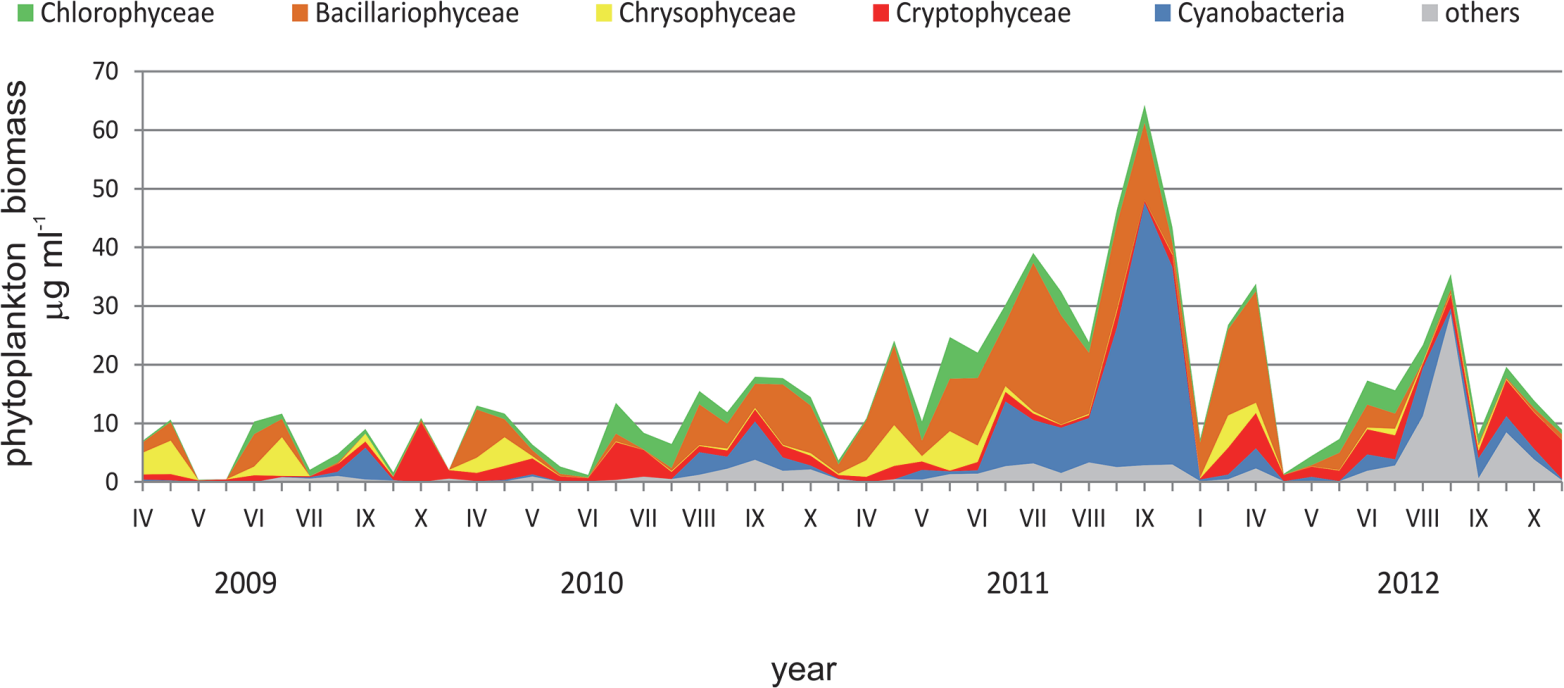
The biomass of phytoplankton groups in the investigated period (an example from the depth of 1 m).

The lowest values of phytoplankton biomass in subsequent years were found in the first year of the study and the highest in 2011 (Fig 7). These values ranged from 0.2 to 11.7 μg ml^- 1^ in 2009, 1.2–34.4 μg ml^- 1^ in 2010, 10.3–66.0 μg ml^- 1^ in 2011 and 3.9–49.5 μg ml^- 1^ in 2012. The groups constituting the highest biomass of phytoplankton were diatoms, cryptophytes and cyanobacteria.

Phytoplankton was also divided into two groups based on the possibility of its consumption by zooplankton: nonedible microplankton and edible nanoplankon. Microplankton abundance varied from 70.0 to 58.0 10^3^ spec. in 1 ml. Nanoplankton was less abundant and ranged from 52 to 25.7 spec. ml^-1^. On the other hand, in the biomass of these two size fractions, microplankton ranged from 0.15 to the value of 61.74 μg ml^- 1^, while nanoplankton ranged from 0.02 to 9.40 μg ml^—1^.

The differences of the values of abundances and biomass of phytoplankton from the four studied years were statistically significant (ANOVA Kruskal-Wallis test, p < 0.01).

### Zooplankton

**Fig 8:**
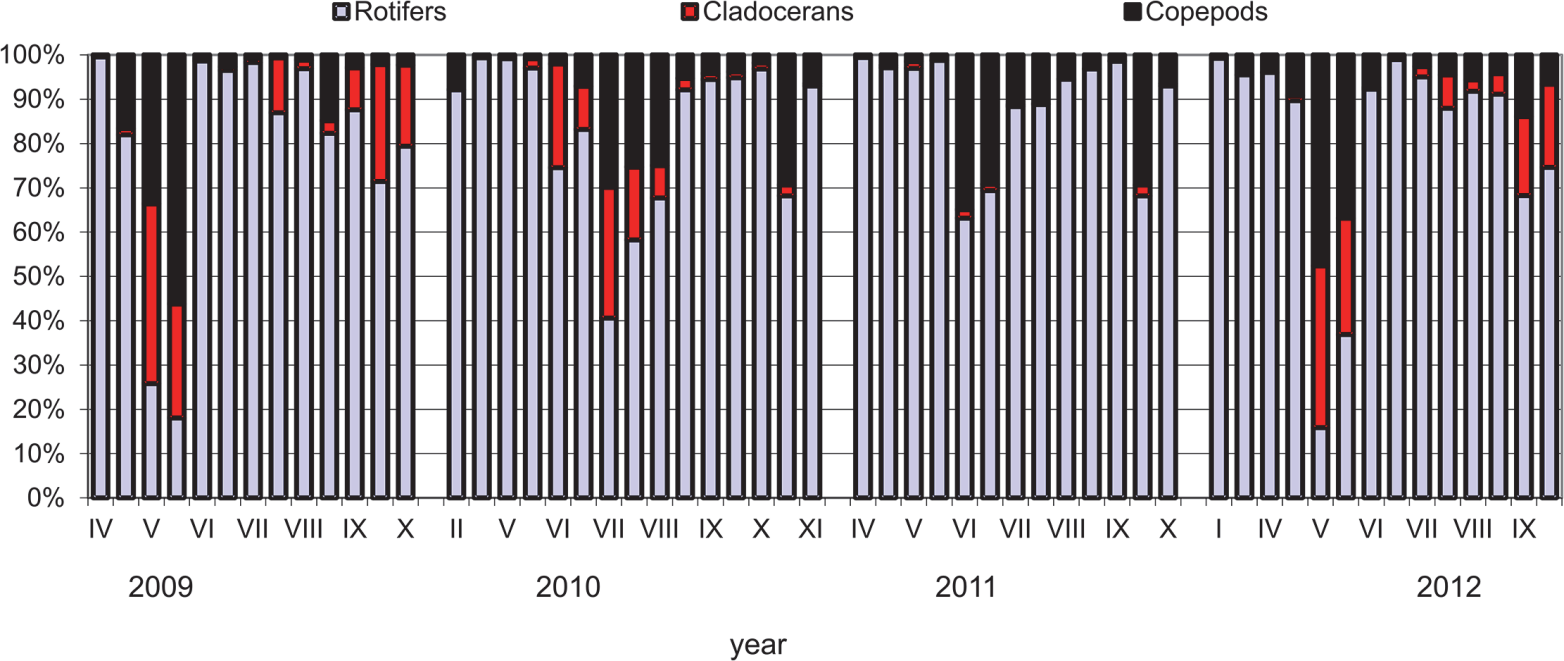
Share of the abundance of zooplankton groups in Maltański Reservoir (an example from the depth of 2 m).

143 zooplankton taxa were determined in total of which 97 were rotifers, 28 cladocerans and 18 copepods. Rotifers were the most abundant zooplankton group, they amount to even more than 99% in the abundance. The share of copepods and cladocerans was smaller, 48% and 40%, respectively (Fig 8). The abundance of filter feeders changed in subsequent years. The lowest abundance of cladorerans was found in 2011. Rotifer abundance increased in each growing season, reaching two peaks: in May or June and August or September. Throughout the four-year period the highest number of rotifers was found in September 2011 (more than 25 10^3^ spec. l^-1^). The most numerous taxa were *Synchaeta* sp. (especially in September 2009), *Keratella cochlearis* Gosse and *K*. *cochlearis* f. *tecta*, *Polyarthra dolichoptera* Idelson, *K*. *quadrata* O. F. Müller, *Anuraeopsis fissa* Gosse (September 2010, 2011, 2012), *Brachionus angularis* Gosse (in June 2011), *Trichocerca pusilla* Lauterborn and *T*. *ruosseleti* Voigt (June 2012), *Filinia terminalis* Plate (May 2010). The most important respecting biomass of rotifers were *Synchaeta pectinata* Ehrenberg, *Polyarthra dolichoptera*, *Asplanchna priodonta* Gosse, *Keratella quadrata* Müller and *Filinia longiseta* Ehrenberg.

**Fig 9:**
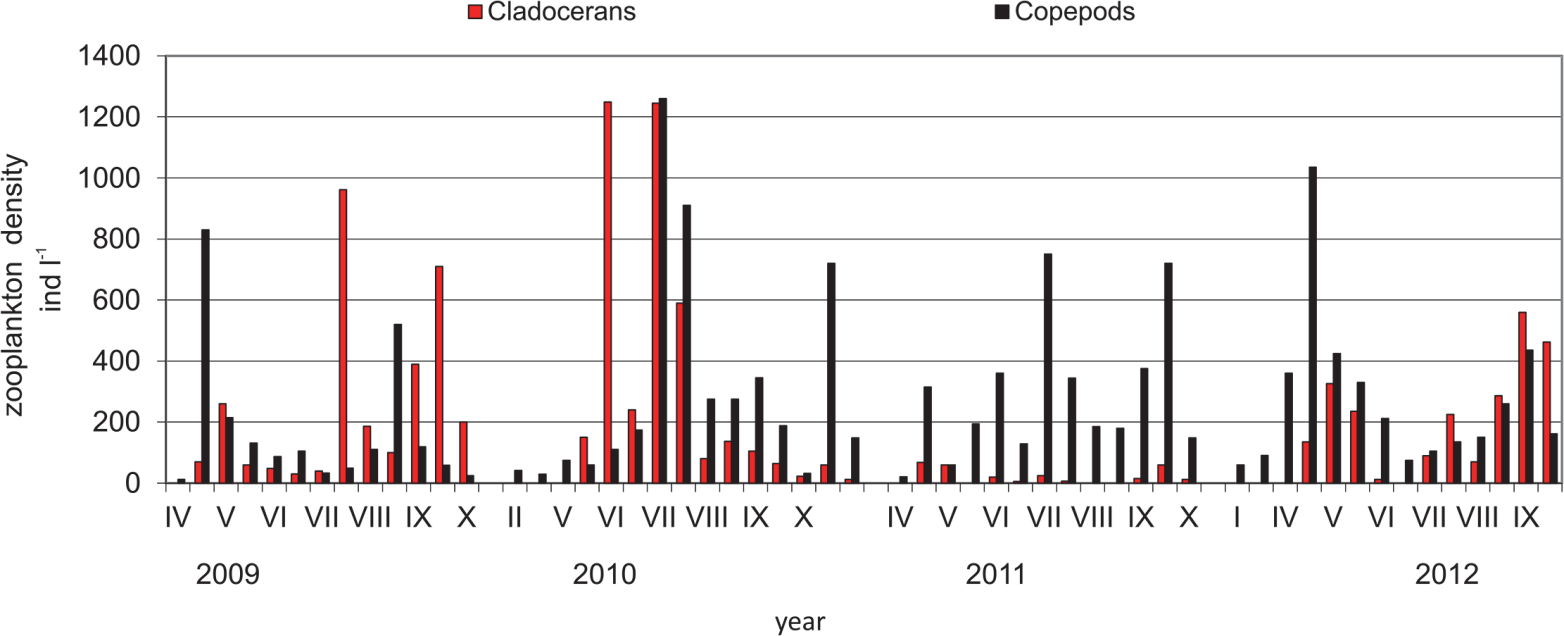
The abundance of cladocerans and copepods (an example from the depth of 2 m).

The average abundance of cladocerans and copepods in the first two years of the study was at a similar level (approximately 100 spec. l^-1^). There was a significant decrease in their abundance in 2011 and the presence of cladocerans in the water column was recorded only sporadically (Figs 8 and 9). In contrast, in 2011 copepods developed quite frequently, their average annual value was even higher than in previous years. Cladocerans were recorded again in large numbers in 2012 and their average abundance in this year increased significantly compared to the previous year.

The most abundant cladoceran species were *Bosmina longirostris*, *B*. *coregoni*, *Daphnia cucullata* (especially from June to September 2010 and 2012), *D*. *hyalina* (May 2009), *Chydorus* sp. (August 2012), *Ch*. *sphaericus* (August to September 2012), *Diaphanosoma brachyurum* (August 2011), *Daphnia longispina* and *D*. *similis* (May 2009 and June 2012). Cladocerans were rather rare in 2011, neither *D*. *similis* nor *D*. *longispina* nor *D*. *hyalina* were noted then.

Among the most frequently represented copepod taxa were: *Mesocyclops leuckarti* (April 2012, August 2010), *Thermocyclops oithonoides* (most often listed in the period from July to September in all four years of study), *Acanthocyclops vermicularis* (July-September 2011) *Acanthocyclops* sp. and *Cyclops vicinus*. Filter feeding copepods such as *Eudiaptomus* e.g. *E*. *gracilis* were recorded mainly in the period from April to May 2009. Their presence in the remaining years was only sporadic.

Cladocerans such as *Daphnia cucullata*, *D*. *similis*, *D*. *hyalina D*. *longispina* and a predatory species *Leptodora kindtii* had the largest share in the total biomass. Among the copepods, juvenile forms were of the highest biomass, i.e. copepodites and *Cyclops furcifer* and *C*. *vicinus*.

### Phytoplankton vs. variables directly connected with restoration (zooplankton filter feeders, zooplankton predators, NH_4_-N, PO_4_-P)

**Fig 10:**
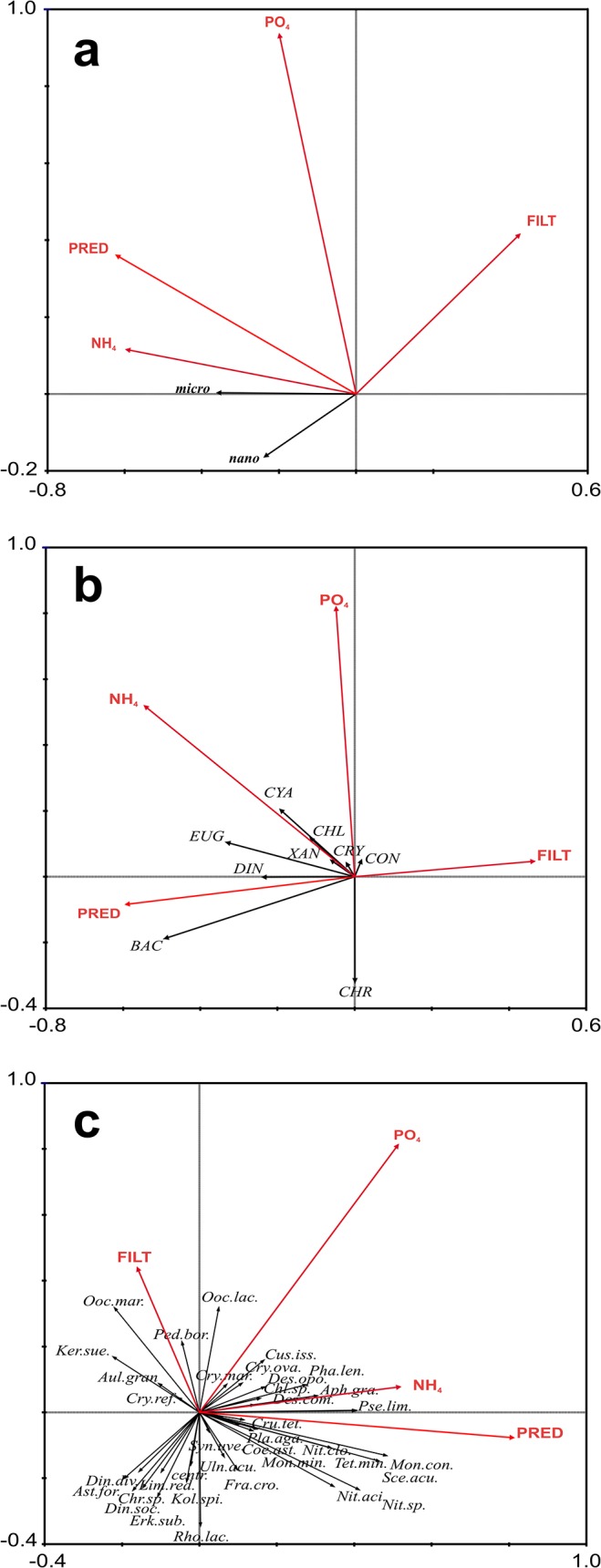
RDA biplot showing relationships between phytoplankton size fractions (a), taxonomical groups (b), the most abundant taxa and selected environmental factors directly dependent on restoration measures (c). Abbreviations: cond-conductivity, ON-organic nitrogen, oxy-oxygen saturation, temp-temperature, R+n—rotifers and nauplii, FILT- filter feeders, PRED- predators, nono- nanophytoplankton, micro-microphytoplankton, *Aph*.*gra*.*-Aphanizomenon gracile*, *Ast*.*for*.*-Asterionella formosa*, *Aul*.*gran*.*-Aulacoseira granulata*, *centr- centric diatom*, *Chr*.*min*.*-Chrysococcus minutus*., *Chl*.*sp*.*-Chlamydomonas sp*., *Coe*.*ast*.*- Coelastrum astroideum*, *Cry*.*mar*.*- Cryptomonas marssonii*, *Cry*. *ova*.*- Cryptomonas ovata*, *Cry*.*ref*.*- Cryptomonas reflexa*, *Cus*.*iss*.*-Cuspidothrix issatschenkoi*, *Cru*. *tet*.*- Crucigenia tetrapedia*, *Des*.*com*.*- Desmodesmus communis*, *Des*.*opo*.*- Desmodesmus opoliensis*, *Din*.*div*.*- Dinobryon divergens*, *Erk*.*sub*.*-Erkenia subaequiciliata*, *Fra*.*cro*.*-Fragilaria crotonensis*, *Kol*.*spi*.*-Koliella spiculiformis*, *Lim*.*red-Limnothrix redekei*, *Mon*.*con*.*-Monoraphidium contortum*, *Mon*. *min*.*- Monoraphidium minutum*, *Nit*.*aci*.*- Nitszchia acicularis*, *Nit*.*clo*.*- Nitszchia acicularis var*. *closterioides*, *Nit*. *spp—Nitszchia spp*., *Ooc*.*lac*.*- Oocystis lacustris*, *Ped*.*bor*.*- Pediastrum boryanum*, *Pha*.*lent*.*- Phacotus lenticularis*, *Pla*.*aga—Planktothrix agardhii*, *Rho*.*lac*.*- Rhodomonas lacustris*, *Sce*.*acu*.*- Scenedesmus acuminatus*, *Syn*.*uve*.*- Synura uvella*, *Uln*.*acu*.*- Ulnaria acus*.

**Fig 11:**
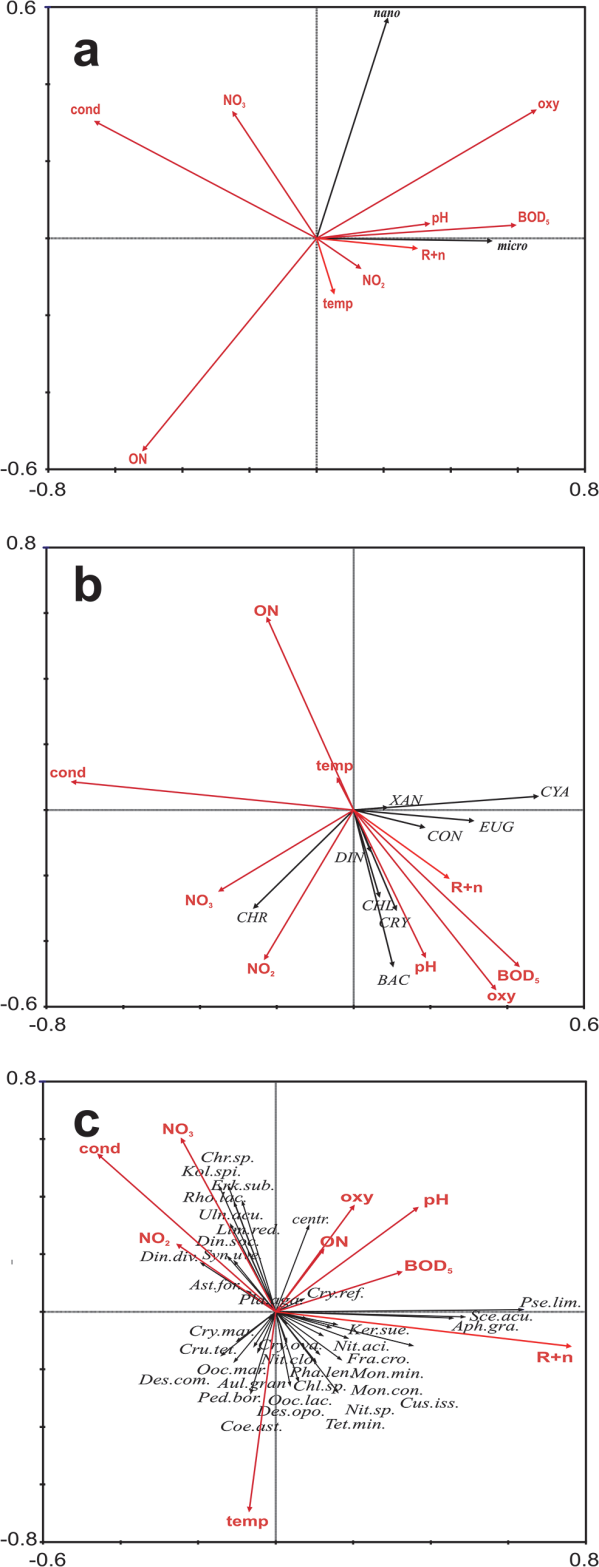
RDA biplot showing relationships between phytoplankton size fractions (a), taxonomical groups (b), the most abundant taxa and selected factors independent from the restoration measurments (c). Abbreviations the same as on Fig 10.

The ordination diagram of RDA showed relationships between the biomass of phytoplankton size fractions and taxonomic groups versus selected environmental factors and zooplankton grazing pressure, directly dependent on restoration measures (Figs 10 and 11). Both nano- and microphytoplankton were negatively correlated with zooplankton filter feeders and positively with zooplanktonic predators and NH_4_-N. No significant correlation between nano- and microphytoplankton and PO_4_-P was noted. The 4 variables explain only 13.1% of variance in the studied relationships. The Monte Carlo test showed that not all the factors were equally important (Fig 10A and Table 2).

**Table 2:** Forward selection results of Monte Carlo test of relationships between phytoplankton (size fractions (a), taxonomical groups (b), the most abundant taxa (c)) and selected environmental factors directly dependent on restoration measures.

	Variable	LambdaA	P	F
a	**nano-and microplankton**			
	**PRED**	0.05	0.002	10.85
	**FILT**	0.05	0.008	10.06
	**NH**_**4**_	0.03	0.008	7.77
	**PO**_**4**_	0.00	0.658	0.20
b	**taxonomical groups**			
	**NH**_**4**_	0.04	0.004	7.58
	**PRED**	0.02	0.006	5.32
	**FILT**	0.04	0.006	8.12
	**PO**_**4**_	0.03	0.006	6.83
c	**the most abundant taxa**			
	**PRED**	0.10	0.002	22.28
	**FILT**	0.02	0.030	4.96
	**NH**_**4**_	0.02	0.024	5.24
	**PO**_**4**_	0.02	0.054	3.51

Abbreviations: FILT- filter feeders, PRED- predators.

Phytoplankton individual taxonomic groups were negatively correlated with zooplankton filter feeders. Such groups as diatoms, euglenophytes and dinophytes were positively correlated with zooplankton predators and NH_4_-N. Cyanobacteria and chlorophytes in particular were positively correlated with PO_4_-P. The 4 variables explain 12.8% of variance in the studied relationships. The Monte Carlo test showed that all 4 factors were statistically significant (Fig 10B and Table 2B).

Individual taxa were also influenced by nutrients and zooplankton. For example, species belonging to cyanobacteria: *Aphanizomenon gracile*, *Cuspidothrix issatschenkoi*, *Planktothrix agardhii* and *Pseudanabaena limnetica* were positively correlated with NH_4_-N and PO_4_-P and zooplankton predators (Fig 10C and Table 2C). Most of the taxa were negatively correlated with zooplankton filter feeders but such green algae as *Oocystis lacustris*, *Pediastrum boryanum* and *Oocystis marssonii* were positively correlated with filter feeders. Small sized *Tetraedron minimum*, *Rhodomonas lacustris*, and bigger *Scenedesmus acuminatus*, *Koliella spiculiformis*, *Fragilaria crotonensis*, *Monoraphidium contortum*, *Nitzschia acicularis* were negatively correlated with zooplankton filter feeders. The ordination diagram of RDA explains 15.8% of variance of phytoplankton taxa.

### Phytoplankton vs. environmental variables independent of the restoration measures (rotifers+nauplii, temperature, conductivity, oxygen saturation, BOD_5_, organic nitrogen, NO_3_-N and NO_2_-N)

Nano- and microphytoplankton were positively correlated with BOD_5_ and water oxygen saturation and negatively with conductivity. These size groups of phytoplankton were not correlated with smaller zooplankton (rotifers+nauplii), temperature, pH, organic nitrogen, NO_3_-N and NO_2_-N (p>0,05, Fig 11A and Table 3A). The ordination diagram of RDA explains 27.9% of variance of the phytoplankton size groups.

**Table 3:** Forward selection results of Monte Carlo test of relationships between a-phytoplankton size fractions, b-taxonomical groups, c-the most abundant taxa and selected environmental factors independent from restoration measurements.

	Variable	LambdaA	P	F
a	**nano-and microplankton**			
	**cond**	0.12	0.002	27.42
	**oxy**	0.11	0.002	28.00
	**BOD**_**5**_	0.02	0.010	6.90
	**NO**_**3**_	0.01	0.064	2.89
	**ON**	0.01	0.170	1.75
	**NO**_**2**_	0.00	0.284	1.32
	**R+n**	0.01	0.458	0.48
	**temp**	0.00	0.462	0.51
	**pH**	0.00	0.950	0.02
b	**taxonomical groups**			
	**cond**	0.08	0.002	18.22
	**oxy**	0.04	0.002	9.00
	**ON**	0.03	0.002	6.37
	**pH**	0.02	0.014	4.87
	**NO**_**3**_	0.02	0.014	4.74
	**temp**	0.02	0.014	4.58
	**BOD**_**5**_	0.02	0.020	4.67
	**NO**_**2**_	0.01	0.060	2.78
	**R+n**	0.00	0.338	1.10
c	**the most abundant taxa**			
	**R+n**	0.20	0.002	51.60
	**cond**	0.04	0.002	9.74
	**ON**	0.03	0.002	8.12
	**BOD**_**5**_	0.04	0.008	10.24
	**temp**	0.03	0.002	9.27
	**NO**_**2**_	0.02	0.002	7.44
	**NO**_**3**_	0.01	0.076	2.74
	**oxy**	0.01	0.142	2.09
	**pH**	0.00	0.310	1.06

Taxonomical groups e.g. diatoms, chrysophytes and cryptophytes were negatively correlated with organic nitrogen, conductivity and temperature. Chrysophytes were positively correlated with NO_2_-N. The ordination diagram of RDA explains 24.1% of variance of the phytoplankton taxonomical groups. Taxonomical groups of phytoplankton were not correlated with NO_3_-N and smaller zooplankton (p>0.05, Fig 11B and Table 3B).

Most of the considered taxa of phytoplankton were positively correlated with the temperature e.g. Coelastrum astroideum, Tetraedron minimum, Desmodesmus opoliensis, Oocystis lacustris. Other taxa: *Erkenia subaequiciliata*, *Kolliella spiculiformis*, *Rhodomonas lacustris* were negatively correlated with the temperature. Cyanobacteria such as *Aphanizomenon gracile*, *Pseudanabaena limnetica*, *Cuspidothrix issatschenkoi* were positively correlated with small zooplankton. Phytoplankton taxa did not correlate with pH and oxygen saturation (p>0,05, Fig 11C and Table 3C). The ordination diagram of RDA explains 38.0% of variance of the phytoplankton taxa.

## Discussion

Phytoplankton is the first biological community to respond to environmental changes. Therefore, it was expected that as a result of MR restoration treatment a low density of phytoplankton and the disappearance of cyanobacteria bloom, which disqualified the reservoir as a recreational facility, would be achieved. Indeed, both seasonal changes of species composition and abundance in the analysed period as well as differences between years have been confirmed. Changes in the quantity and quality of the composition of phytoplankton in the four years of the study were significantly related to both restoration methods, biomanipulation and P-inactivation. As a consequence of the application of two methods of restoration, the abundance and biomass of phytoplankton was low, especially in the first two years following the refilling of the reservoir. Water transparency in the past had always been highest in the first year after the reservoir filling because the abundance of planktivorous fish was low [17]. As the reservoir was stocked with predatory fish, effectiveness of biomanipulation was also strongest at that time. The maximum abundance in the subsequent years of the study did not exceed 29.0 ·10^3^ spec. ml^-1^ in 2009, 14.2·10^3^ spec. ml^-1^ in 2010 and 10.6 ·10^3^ spec. ml^-1^ in 2012, while in the past it often exceeded 30.0 ·10^3^ spec. ml^-1^ [25].

The decrease of phytoplankton abundance and its structural changes were certainly influenced by biomanipulation. Phytoplankton dynamics were strictly connected with the abundance and taxonomic composition of zooplankton (negative and positive correlations). The growth of nanophytoplankton and most of the phytoplankton taxonomical groups, especially the most abundant green algae, diatoms and cyanobacteria, were effectively limited by zooplankton filter feeders, particularly large cladocerans observed in the years 2009, 2010 and 2012 (negative correlation), particularly in May and June. A similar phenomenon of a high abundance of cladocerans, an increased abundance of *Daphnia* spp. and an increased zooplankton/phytoplankton ratio has been reported from other restored lakes [35–36]. Only in the third year of the study was phytoplankton density in the MR high, reaching a maximum of 60 thousand spec. ml^-1^. The probable explanation was that a large number of planktivorous fish entered the reservoir with the river water. During that time draining water from a preliminary reservoir located upstream took place [10]. The pressure of planktivorous fish must have been so high that in 2011 large filter feeders were absent (especially *D*. *similis*, *D*. *longispina* and *D*. *hyalina*) and the numbers of other daphnids were lowest in the period of the four-year cycle. The low number of daphnids in 2011 can also be attributed to the intensive growth of cyanobacteria. Many authors point out that the filtration apparatus of *Daphnia* may be clogged by filamentous cyanobacteria [37].

Cyanobacterial taxa are considered to be unattractive food for zooplankton filtrators [34]. On the other hand, the decrease in cladoceran abundance was possibly caused by the inhibitory effect of cyanocacteria due to the production of toxins. *Daphnia* species attempt to stay away from toxic cyanobacteria [38]. It is also worth noting that in an experiment relating to the presence of filamentous cyanobacteria in the water, large-size populations are converted to smaller ones. Among cladocerans of the genus *Daphnia*, *D*. *cucullata* is considered to be the most resistant species. It may be favoured by its lower sensitivity to filament inhibition combined with lower susceptibility to fish predation [39]. This is confirmed by observations in the MR, in which the only species of the genus *Daphnia* in 2011 was *D*. *cucullata*. The fact that *Daphnia* is able to eliminate large bloom-forming cyanobacterial colonies effectively is known from the literature, especially after being released from fish predation [40–41]. This was confirmed by RDA analysis in the present studies, in which Cyanobacteria were negatively correlated with filter feeders, although the relation was not very strong. As an example *Planktothrix agardhii* and *Pseudanabaena limnetica* were negatively correlated with zooplankton filter feeders. These taxa could simultanously even be stimulated by zooplankton predators (positive correlation). Their nutrient excretions may cause an increase in phytoplankton biomass, as stated in Swarzędzkie Lake [42–43].

A positive effect of biomanipulation on lessening the abundance of phytoplankton was demonstrated in the MR in 1993–1996 [17]. It was shown, however, that it is very difficult to achieve a spectacular long-term decline in phytoplankton abundance and biomass using the biomanipulation procedure. There was a so-called feedback effect that significantly reduces the effectiveness of biomanipulation. Therefore, an additional method of restoration was used in MR from 2005, aimed at phosphorus binding from the water column and depositing it in the form of insoluble salts in bottom sediments. The main aim of this chemical treatment was to remove the phosphorus loads available for phytoplankton. This method also failed to achieve a long term decrease of phytoplankton abundance in the first years of its use [25]. In such throughflow lakes, the restoration activity should be continued due to the constant inflow of nutrients from the catchment area (rainwater outlets). Similar situation were noted in other restored lakes eg Lake Rusałka, Swarzędzkie or Uzarzewskie [13, 45]. In those lakes water blooms during the summer and/or autumn were caused by intense external and internal nutrient loading. There were also examples of intense decreases of cyanobacterial abundance in relatively small and isolated lakes such as Trumen (Sweden) [5] or Lake Rauwbraken (The Netherlands)[18]. Cyanobacteria positively correlated with NH_4_-N and PO_4_-P in MR. Both parameters especially influenced the larger phytoplankton (microplankton, positive correlation). This correlation was probably strongly influenced by the data from 2011, when the concentration of ammonium nitrogen clearly increasedExperiments carried out on several lakes in Poland and around the world show that the most effective treatment is achieved by using several methods simultaneously [10]. The use of chemicals (iron sulphate, magnesium chloride and Sinobent) not only results in the precipitation of phosphorus but also adversely affects the phytoplankton. As shown by Budzyńska [44], these chemical substances used in two water bodies affect the cyanobacterial filaments, shortening their lengths. This fact could facilitate their consumption by cladocerans. The chemical treatment probably had the greatest influence on cyanobacteria and green algae which positively correlated with PO_4_-P and NH_4_-N. The case study of Maltański Reservoir has demonstrated that the use of one restoration method was less effective than the simultaneous use of two methods. In 1993–96, when MR was restored with biomanipulation only, phytoplankton abundance was higher and reached 15·10^3^, 34·10^3^, 72·10^3^ and 36·10^3^ spec.ml^-1^ in subsequent years [17,45]. These values were much lower in the present study due to the simultaneous use of the second method of restoration, with the exception of 2011. The use of a chemical treatment causes an immediate effect, although its result is short-term. After a few weeks phytoplankton abundance returns to its former, pretreatment state. Similar experiences with this method have also been reported from other water bodies such as Rusałka Reservoir and Lake Uzarzewskie by Budzyńska [44]. This phenomenon is caused by the external load of nutrients continuously flowing to these lakes with waters from their tributaries.

Statistical analyses indicated the importance of physico-chemical and biological environmental variables to be more significant than variables connected with restoration measures in terms of influencing the abundance of phytoplankton. As expected, it was found that the biomass of different groups of phytoplankton was positively correlated with NO_2_-N, pH, oxygen saturation and BOD_5_ and negatively with conductivity.

Water temperature clearly influenced the development of various groups of phytoplankton. Many taxa, e.g. *Coelastrum astroideum*, *Pediastrum boryanum*, *Desmodesmus opoliensis*, positively correlated with temperature. During the considered period in MR euglenophytes, chlorophytes, dinophytes and xantophytes dominated, especially at higher temperatures, while euglenophytes, cryptophytes, diatoms and chrysophytes dominated at lower temperatures. The dominance of a phytoplankton group is dependent on resistance to grazing, resistance to sedimentation and the ability to perform vertical migration [46]. It is believed that cyanobacteria have the best chance to dominate the phytoplankton at higher water temperatures [47], although some studies do not support the hypothesis that cyanobacteria have higher optimum temperatures for growth and higher growth rates than chlorophytes [48]. There are cyanobacterial species e.g *Planktothrix rubescens*, *P*. *agardhii* or *Woronichiania naegeliana* that cause water blooms in autumn, in low temperatures [49–51]. Cyanobacteria also grew intensively in MR in the autumn, when the temperature dropped. Moreover, other groups of phytoplankton, especially green algae, dominated during the period of higher water temperature. The restoration treatments leading to a decline in phytoplankton abundance and biomass, especially of cyanobacteria, were important there. Due to these treatments the abundance of cyanobacteria was lowest in September 2009, however, their percentage was highest (33%) at the same time. The most important cyanobacteria species were *Pseudanabaena limnetica* and *Cuspidothrix issatschenkoi*. The first belongs to Oscillatoriales and is resistant to shading and water mixing [52]. The second one is a species invasive to Polish algal flora [53–56]. Both species were observed in this reservoir from the seventies [57]. They are strictly related to the availability of PO_4_-P (positive correlation, p = 0.05) and are able to form intense algal blooms [58]. In September 2010, the presence of *Aphanizomenon gracile* was revealed in MR. These taxa are also potentially toxic species [59–60]. According to a study conducted by Mehnert et al. [61], invasive species have a higher growth rate than native species, which may explain the high share of *C*. *issatschenkoi* in the first year of MR research. In turn, after the restoration of Lake Balaton [20, 61] the coexistence of *A*. *gracile* and an invasive species of blue-green algae was reported. Interestingly, during the analysed period the cyanobacterium *Aphanizomenon flos-aquae*, which is a taxon typical of this reservoir and which causes water blooms, was not recorded. This species appeared in the reservoir in previous periods in the first years after filling with water, e.g. in 1993, when biomanipulation treatments were used for the first time in the reservoir [17, 45] and in 2005, when an additional method of remediation—chemical treatment—was applied there [25].

An increasing amount of data is available on the concentration of toxins derived from cyanobacteria in inland waters which are used as a source of drinking water or for recreational purposes [62–65]. Therefore, substantial restoration activities should be undertaken for their elimination or restriction.

Some phytoplankton species can adapt to lower temperatures. The highest participation in the abundance and biomass of phytoplankton in spring was noted in case of diatoms, chrysophytes, cryptophytes and chlorophytes. These groups prefer lower temperatures (negative correlation,). This observation is consistent with the model of plankton succession (PEG) [47]. *Erkenia subaequiciliata*, *Dinobryon sociale*, *Rhodomonas lacustris*, *Koliella spiculiformis and Chrysococcus sp* were the most abundant species. These so-called R-species [66] tolerate low light intensity. Some of them are known to be successful in phosphorus-limited conditions due to their mixotrophy, e.g. *Dinobryon* [67] or *Rhodomonas* [68]. All these species were positively correlated in this study with conductivity, NO_2_-N, organic nitrogen, oxygen saturation and negatively correlated with PO_4_-P concentration. They are species that maintain growth at a low average light level and are tolerant of a well-mixed, poorly insolated environment. They were also under high pressure from zooplankton filter feeders (negative correlation).

Larger phytoplankton taxa, not only cyanobacteria such as *Aphanizomenon gracile*, *Pseudanabaena limnetica*, *Cuspidothrix issatschenkoi*, but also *Nitzschia* sp., *Fragilaria crotonensis*, *Scenedesmus acuminatus*, could also be stimulated by the zooplankton, especially smaller ones (mostly rotifers). Most of the phytoplankton taxa were positively correlated with small zooplankton. The ordination diagram of variables that were not directly influenced by the restoration measures explained 38.0% of variance of the phytoplankton taxa, of which small zooplankton explained 20% of variance (Table 3C).

Factors connected with restoration procedures had less influence than parameters independent of restoration. The ordination diagram of RDA in the case of factors connected with restoration displayed a smaller percentage of variance in the algal size fractions and/or taxa abundance than those independent of restoration. The listed parameters as well as many other factors which were not taken into account in this study affected the quantitative and qualitative composition of the phytoplankton in the reservoir.

The restoration in MR did not cause the spectacular decrease in the phytoplankton abundance. Supplies of nutrients from surface runoff are responsible for that so restoration should be continued in this reservoir.

## References

[1] Gawrońska H, Łopata M, Jaworska B. The effectiveness of the phosphorus inactivation method in reducing the trophy of lakes of different morphometric and hydrological features Limnol. Rev. 2007; 7(1): 27–34.

[2] Jaworska B, Dunalska J, Górniak D, Bowszys M. Phytoplankton dominance structure and abundance as indicators of the trophic state and ecological status of Lake Kortowskie (northeast Poland) restored with selective hypolimnetic withdrawal Arch. Pol. Fish. 2014; 22 (1): 7–15.

[3] Łopata M, Gawrońska H, Jaworska B, Wiśniewski G. Restoration of two shallow, urban lakes using the phosphorus inactivation method—Preliminary results. Water Sci. Technol. 2013; 68 (10): 2127–2135. 10.2166/wst.2013.461 24292458

[4] Kozak A, Gołdyn R. Variation in phyto-and zooplankton of the restored Lake Uzarzewskie. Pol. J. Environ. Stud. 2014; 23 (4): 1201–1209.

[5] Cronberg G Changes in the phytoplankton of Lake Trummen induced by restoration. Hydrobiologia 1982; 86: 185–193.

[6] Cooke GD, Welch EB, Peterson S, Nichols SA. Restoration and Management of Lakes and Reservoirs, Third Edition, Taylor and Francis group; 2003 pp. 1–616.

[7] Łopata M. Gawrońska H. Phosphorus immobilization in Lake Głęboczek following treatment with polyaluminum chloride Oceanol. Hydrobiol. Stud. 2008; 37 (2): 99–105

[8] Gołdyn R, Podsiadłowski S, Kowalczewska-Madura K, Dondajewska R, Szela̧g-Wasielewska E, Budzyńska A, et al Functioning of the Lake Rusałka ecosystem in Poznań (western Poland). Oceanol. Hydrobiol. Stud. 2010; 39 (3): 65–80.

[9] Gołdyn R, Messyasz B, Domek P. Windhorst W., Hugenschmidt C., Nicoara M, et al The response of Lake Durowskie ecosystem to restoration measures. Carpath. J. Earth Environ. Sci. 2013; 8 (3): 43–48.

[10] Gołdyn R, Podsiadłowski S, Dondajewska R, Kozak A. The sustainable restoration of lakes—towards the challenges of the Water Framework Directive. Ecohydrol. Hydrobiol. 2014; 23 (4): 1201–1209.

[11] Grochowska JK, Brzozowska R. The influence of different recultivation methods on the water buffer capacity in a degraded urban lake. Knowledge and Management of Aquatic Ecosystems 2013; 410: Article no 1.

[12] Kowalczewska-Madura K, Gołdyn R, Dondajewska R. Phosphorus release from the bottom sediments of Lake Rusałka (Poznań, Poland Oceanol. Hydrobiol. Stud. 2010; 39 (4): 135–144.

[13] Kozak A, Kowalczewska-Madura K, Gołdyn R, Czart A. Phytoplankton composition and physico-chemical properties in restored Swarzędzkie Lake—preliminary results. Arch. Pol. Fish. 2014; 22: 17–28.

[14] Kozak A, Dondajewska R, Kowalczewska-Madura K, Gołdyn R, Holona T. Water Quality and phytoplankton community in selected recreational lakes and reservoirs under restoration measures in Western Poland. Pol. J. Natur. Sc. 2013; 28 (2): 217–226.

[15] Zamparas M, Zacharias I. Restoration of eutrophic freshwater by managing internal nutrient loads. A review. Sci. Total Environ. 2014; 496: 551–562. 10.1016/j.scitotenv.2014.07.076 25108796

[16] Bishop WM, McNabb T, Cormican I. Willis BE, Hyde S. Operational Evaluation of Phoslock Phosphorus Locking Technology in Laguna Niguel Lake, California. Water Air Soil Pollut. 2014; 225: 2018.

[17] Kozak A, Gołdyn R. Zooplankton versus phyto- and bacterioplankton in the Maltanski Reservoir (Poland) during an extensive biomanipulation experiment J. Plankton Res. 2004; 26 (1): 37–48.

[18] Lürling M, van Oosterhout F. Controlling eutrophication by combined bloom precipitation and sediment phosphorus inactivation. Water Res. 2013; 47: 6527–6537. 10.1016/j.watres.2013.08.019 24041525

[19] Moos MT, Taffs KH, Longstaff BJ, Ginn BK. Establishing ecological reference conditions and tracking post-application effectiveness of lanthanum-saturated bentonite clay (Phoslock) for reducing phosphorus in aquatic systems: An applied paleolimnological approach. J. Environ. Manage. 2014; 141: 77–85. 10.1016/j.jenvman.2014.02.038 24768837

[20] Padisak J, Reynolds CS. Selection of phytoplankton associations in Lake Balaton, Hungary, in response to eutrophication and restoration measures, with special reference to the cyanoprokaryotes. Hydrobiologia 1998; 384: 41–53.

[21] Shapiro J, Lamarra V, Lynch M. Biomanipulation, an ecosystem approach to lake restoration., In Brezonik P.L. J.L Fox (Eds.), Proceedings Symposium on Water quality management through biological control. Univ. Florida Gainsville; 1991: 85–96.

[22] Gołdyn R, Kozak A, Romanowicz W. Food-web manipulation in the Maltański Reservoir. Hydrobiologia, 1997; 342–343: 327–333.

[23] Benndorf J. Conditions for effective biomanipulation; conclusions derived from whole-lake experiments in Europe. Hydrobiologia, 1990; 200–201 (1): 187–203.

[24] Jeppesen E, Jensen JP, Søndergaard M, Lauridsen T, Pedersen LJ, Jensen L. Top-down control in freshwater lakes: The role of nutrient state, submerged macrophytes and water depth. Hydrobiologia, 1997; 342–343: 151–164.

[25] Kozak A. Phytoplankton in the restored Maltański Reservoir in the years 2005–2006. Teka Kom. Ochr. Kszt. Środ. Przyr. 2007; 4: 7–13.

[26] Elbanowska H, Zerbe J, Siepak J. Physico-chemical water analyses [Fizyczno-chemiczne badanie wód] (in Polish). UAM Publ., Poznań; 1999.

[27] Wetzel RG, Likens GE. Limnological Analyses. Springer Verlag, New York 1991.

[28] Bottrell HM, Duncan A, Gliwicz ZM, Grygierek A, Herzig A, Hillbricht-Ilkowska H, et al A review of some problems in zooplankton production studies—Norw J. Zool. 1976; 24: 419–456.

[29] Ejsmont-Karabin J. Empirical equations for biomass calculation of planktonic rotifers, Pol. Arch. Hydrobiol. 1998; 45: 513–522.

[30] Radwan S (ed.). Freshwater fauna of Poland [Fauna słodkowodna Polski] (in Polish). 32 A, B., Polskie Towarzystwo Hydrobiologiczne, Uniwersytet Łódzki, Oficyna Wydawnicza Tercja, Łódź; 2004.

[31] Shannon CE, Weaver W. The Mathematical Theory of Communication, University of Illinois Press, Urbana; 1949.

[32] ter Braak CJF, Smilauer P. CANOCO reference manual and CanoDraw for Windows user's guide: software for canonical community ordination (version 4.5) TNO Institute of Applied Computer Science, Wageningen; 2002.

[33] Knoechel R, Holtby LB. Cladoceran filtering rate: body length relationship for bacterial and large algal particles. Limnol. Oceanogr. 1986; 31: 195–200.

[34] Lampert W. The relationship between zooplankton biomass and grazing: a review. Limnologica (Berlin) 1988; 19: 11–20.

[35] Chen F, Shu T, Jeppesen E, Liu Z, Chen Y. Restoration of a subtropical eutrophic shallow lake in China: Effects on nutrient concentrations and biological communities. Hydrobiologia 2013; 718 (1): 59–71

[36] Jeppesen E, Søndergaard M, Meerhoff M, Lauridsen TL, Jensen JP. Shallow lake restoration by nutrient loading reduction—Some recent findings and challenges ahead. Hydrobiologia, 2007; 584 (1): 239–252.

[37] Bednarska A, Dawidowicz P. Change in filter-screen morphology and depth selection: Uncoupled responses of Daphnia to the presence of filamentous cyanobacteria. Limnol. Oceanogr. 2007; 52 (6): 2358–2363.

[38] Forsyth DJ, James M R, Cryer M. Alteration of seasonal and dial patterns in vertical migration of zooplankton by Anabaena and planktivorous fish. Arch. Hydrobiol. 1990; 117: 385–404.

[39] Hawkins P, Lampert W. The effect of Daphnia body size on filtering rate inhibition in the presence of a filamentous Cyanobacterium. Limnol.Oceanogr. 1989; 34(6): 1084–1089.

[40] Kurmayer R. Competitive ability of Daphnia under dominance of non-toxic filamentous cyanobacteria. Hydrobiologia 2001; 442: 279–289.

[41] Sarnelle O. Initial conditions mediate the interaction between Daphnia and bloom-forming cyanobacteria. Limnol. Oceanogr. 2007; 52 (5): 2120–2127.

[42] Gołdyn R, Kowalczewska-Madura K. Interactions between phytoplankton and zooplankton in the hypertrophic Swarzȩdzkie Lake in western Poland. J. Plankton Res. 2008; 30 (1): 33–42.

[43] Kowalczewska-Madura K, Gołdyn R. Models of phosphorus turn-over in a hypertrophic Lake: The Lake Swarzȩdzkie case study. Oceanol. Hydrobiol. Stud. 2010; 39 (3): 21–33.

[44] Budzyńska A. Changes in the phytoplankton of two lakes under the influence of iron treatment restoration. [Zmiany w fitoplanktonie dwóch jezior pod wpływem rekultywacji z użyciem siarczanu żelaza] (in Polish). PhD thesis, Adam Mickiewicz University; 2010.

[45] Kozak A. Seasonal changes occurring over four years in a reservoir's phytoplankton composition. Pol. J. Environ. Stud., 2005; 14 (4): 451–465.

[46] Sommer U, Gliwicz ZM, Lampert W, Duncan A. The PEG-model of succession of planktonic events in fresh waters. Arch. Hydrobiol. 1986; 106: 433–471.

[47] Elliott JA. Is the future blue-green? A review of the current model predictions of how climate change could affect pelagic freshwater cyanobacteria. Water Res. 2012; 46 (5): 1364–1371. 10.1016/j.watres.2011.12.018 22244968

[48] Lürling M, Eshetu F, Faassen EJ, Kosten S, Huszar VLM. Comparison of cyanobacterial and green algal growth rates at different temperatures. Freshwater Biology 2013; 58: 552–559. 10.1016/j.archoralbio.2012.06.001 22749497

[49] Wilk-Woźniak E, Mazurkiewicz-Boroń G. The autumn dominance of cyanoprokaryotes in a deep meso-eutrophic submontane reservoir. Biologia 2003; 58, 1: 17–24

[50] Walsby A E, Schanz F, Schmid M. The Burgundy-blood phenomenon: A model of buoyancy change explains autumnal waterblooms by Planktothrix rubescens in Lake Zürich. New Phytol. 2006; 169, 1: 109–122. 16390423 10.1111/j.1469-8137.2005.01567.x

[51] Pawlik-Skowrońska B, Toporowska M. Blooms of toxin-producing Cyanobacteria—A real threat in small dam reservoirs at the beginning of their operation. Oceanol. Hydrobiol. Stud. 2011; 40, 4: 30–37.

[52] Mischke U. Cyanobacteria associations in shallow polytrophic lakes: influence of environmental factors. Acta Oecol. 2003; 24: 11–23.

[53] Stefaniak K, Kokocinski M. Occurrence of invasive Cyanobacteria species in polimictic lakes of the Wielkopolska region (western Poland). Oceanol. Hydrobiol. Stud., 2005; 34(Suppl. 3), 137–148.

[54] Pełechata A, Pełechaty M, Pukacz A. Cyanoprokaryota of shallow lakes of Lubuskie Lakeland (mid-western Poland). Oceanol. Hydrobiol. Stud. 2006; 35, 1: 3–14.

[55] Kobos J, Błaszczyk A, Hohlfeld N, Toruńska-Sitarz A, Krakowiak A, Hebel A, et al Cyanobacteria and cyanotoxins in Polish freshwater bodies. Oceanol. Hydrobiol. Stud. 2013; 42(4):358–378.

[56] Wilk-Woźniak E, Najberek K. Towards clarifying the presence of alien algae in inland waters—can we predict places of their occurrence? Biologia 2013; 68/5: 838–844

[57] Stefko B. Fitoplankton Jeziora Maltańskiego. In Dąmbska, I. (ed.) Fitoplankton sztucznych jezior położonych na terenie Poznania [Phytoplankton of manmade lakes located in Poznań] (in Polish). PTPN, Prace Kom. Biol. 1976; 42: 55–117.

[58] Rojo C, Alvarez Cobelas M. Population dynamics of Limnothrix redekei, Oscillatoria lanceaeformis, Planktothrix agardhii and Pseudanabaena limnetica (cyanobacteria) in a shallow hypertrophic lake (Spain). Hydrobiologia, 1994; 275–276 (1): 165–171.

[59] Kopp R, Mareš J, Palíková M, Navrátil S, Kubíček Z, Ziková A, et al Biochemical parameters of blood plasma and content of microcystins in tissues of common carp (*Cyprinus carpio* L.) from a hypertrophic pond with cyanobacterial water bloom: Review Article, Aquaculture Res. 2009; 40 (15): 1683–1693.

[60] Hodoki Y, Ohbayashi K, Kobayashi Y Okuda N, Nakano I. Detection and identification of potentially toxic cyanobacteria: Ubiquitous distribution of *Microcystis aeruginosa* and *Cuspidothrix issatschenkoi* in Japanese lakes. Harmful Algae 2012; 16: 9.

[61] Mehnert G, Leunert F, Cirés S, Jöhnk D, Rücker J, Nixdorf B, et al Competitiveness of invasive and native cyanobacteria from temperate freshwaters under various light and temperature conditions. J. Plankton Res. 2010; 32 (7): 1009–1021.

[62] Ballot A, Fastner J, Lentz M, Wiedner C. First report of anatoxin-a-producing cyanobacterium *Aphanizomenon issatschenkoi* in northeastern Germany. Toxicon 2010; 56 (6): 964–971. 10.1016/j.toxicon.2010.06.021 20615427

[63] Zagajewski P, Gołdyn R, Fabiś M. Water blooms and their toxicity in public swimming areas of lakes in the Poznań district. Oceanol. Hydrobiol. Stud. 2007; 36 (1): 181–187.

[64] Zagajewski P, Gołdyn R, Fabiś M. Cyanobacterial volume and microcystin concentration in recreational lakes (Poznań —Western Poland). Oceanol. Hydrobiol. Stud. 2009; 38 (2): 113–120.

[65] Kokociński M, Mankiewicz-Boczek J, Jurczak T, Spoof L, Meriluoto J, Rejmonczyk E, et al *Aphanizomenon gracile* (Nostocales), a cylindrospermopsin-producing cyanobacterium in Polish lakes. Environ. Sci. Pollut. Res. 2013; 20 (8): 5243–5264. 10.1007/s11356-012-1426-7 PMC3713259 23378259

[66] Reynolds CS, Huszar V, Kruk C, Naselli-Flores L, Melo S. Towards a functional classification of the freshwater phytoplankton. J. Plankton Res. 2002; 24 (5): 417–428.

[67] Kangro K, Laugaste R, Nõges P, Ott I. Long-term changes and seasonal development of phytoplankton in a strongly stratified, hypertrophic lake In: I. Ott and T. Kõiv (eds), Lake Verevi, Estonia—A Highly Stratified Hypertrophic Lake. Hydrobiologia 2005; 547: 91–10.

[68] Hammer A, Schumann R, Schubert H. Light and temperature acclimation of *Rhodomonas salina* (cryptophyceae): Photosynthetic performance. Aquat. Microb. Ecol. 2002; 29 (3): 287–296.

